# mRNA Structural Constraints on EBNA1 Synthesis Impact on *In Vivo* Antigen Presentation and Early Priming of CD8^+^ T Cells

**DOI:** 10.1371/journal.ppat.1004423

**Published:** 2014-10-09

**Authors:** Judy T. Tellam, Jie Zhong, Lea Lekieffre, Purnima Bhat, Michelle Martinez, Nathan P. Croft, Warren Kaplan, Ross L. Tellam, Rajiv Khanna

**Affiliations:** 1 QIMR Centre for Immunotherapy and Vaccine Development and Tumour Immunology, QIMR Berghofer Medical Research Institute, Brisbane, Queensland, Australia; 2 Medical School, Australian National University, Canberra, Australian Capital Territory, Australia; 3 Department of Biochemistry and Molecular Biology, Monash University, Clayton, Victoria, Australia; 4 Peter Wills Bioinformatic Centre, Garvan Institute of Medical Research, Sydney, New South Wales, Australia; 5 CSIRO Agriculture Flagship, Commonwealth Scientific and Industrial Research Organization, Brisbane, Queensland, Australia; University of Southern California Keck School of Medicine, United States of America

## Abstract

Recent studies have shown that virally encoded mRNA sequences of genome maintenance proteins from herpesviruses contain clusters of unusual structural elements, G-quadruplexes, which modulate viral protein synthesis. Destabilization of these G-quadruplexes can override the inhibitory effect on self-synthesis of these proteins. Here we show that the purine-rich repetitive mRNA sequence of Epstein-Barr virus encoded nuclear antigen 1 (EBNA1) comprising G-quadruplex structures, limits both the presentation of MHC class I-restricted CD8^+^ T cell epitopes by CD11c^+^ dendritic cells in draining lymph nodes and early priming of antigen-specific CD8^+^ T-cells. Destabilization of the G-quadruplex structures through codon-modification significantly enhanced *in vivo* antigen presentation and activation of virus-specific T cells. *Ex vivo* imaging of draining lymph nodes by confocal microscopy revealed enhanced antigen-specific T-cell trafficking and APC-CD8^+^ T-cell interactions in mice primed with viral vectors encoding a codon-modified EBNA1 protein. More importantly, these antigen-specific T cells displayed enhanced expression of the T-box transcription factor and superior polyfunctionality consistent with the qualitative impact of translation efficiency. These results provide an important insight into how viruses exploit mRNA structure to down regulate synthesis of their viral maintenance proteins and delay priming of antigen-specific T cells, thereby establishing a successful latent infection *in vivo*. Furthermore, targeting EBNA1 mRNA rather than protein by small molecules or antisense oligonucleotides will enhance EBNA1 synthesis and the early priming of effector T cells, to establish a more rapid immune response and prevent persistent infection.

## Author Summary

Maintenance proteins of viruses establishing latent infections regulate their synthesis to levels sufficient for maintaining persistent infection but below threshold levels for host immune detection. The Epstein-Barr virus maintenance protein, EBNA1, has recently been shown to contain unusual G-quadruplex structures within its repeat mRNA that reduces its translational efficiency. In this study we assess how modification of the EBNA1 mRNA repeat sequence to destabilize the native G-quadruplex structures and thereby increase translation, impacts on the activation of EBNA1-specific T cells *in vivo*. Mice primed with viral vectors encoding a more efficiently translated EBNA1 mRNA revealed increased trafficking of EBNA1-specific T cells, an enhanced functional profile and increased expression of transcription factors providing evidence for a potential link between mRNA translational efficiency and antigen presentation *in vivo* and the resultant impact on the functional programming of effector T cells. These findings suggest a novel approach to therapeutic development through the use of antisense strategies or small molecules targeting EBNA1 mRNA structure.

## Introduction

The interaction of a peptide-MHC class I (pMHC-I) complex on antigen presenting cells (APCs) with a T cell receptor (TCR) on CD8^+^ T cells, initiates the activation of antigen-specific CD8^+^ T cells [1]. Recent *in vitro* studies from many groups have revealed that endogenously processed MHC class I-restricted epitopes are predominantly generated from rapidly degraded defective ribosomal products (DRiPs) rather than from the degradation of full-length, stable viral proteins [2], [3], [4], [5], [6]. This process suggests that by regulating the production of antigen or DRiPs in host cells during viral infection we could beneficially influence the generation and presentation of MHC class I-restricted epitopes and the induction of antigen-specific immune responses. Indeed, earlier studies by Ryan and colleagues have shown that the magnitude of CD8^+^ T cell activation during mycobacterial infection is determined by the level of antigen first encountered by naïve T cells [7]. Furthermore, modulation of antigen expression by slowly replicating pathogens may facilitate their persistence by delaying the development of acquired immune responses [8], [9].

Epstein-Barr virus (EBV) is a classic example of a persistent infection in which down-regulation of viral protein synthesis limits antigen presentation to CD8^+^ T cells through the MHC class I pathway. EBV encoded nuclear antigen 1 (EBNA1) is a critical viral genome maintenance protein expressed in all EBV-associated malignancies. Constraints on EBNA1 self-synthesis limit the presentation of T cell epitopes on the surface of virus-infected cells [10], [11]. Extensive studies have shown that this restricted presentation is due in part to an internal glycine-alanine repeat (GAr) domain within EBNA1 [12], [13], [14]. Although it has been reported that the GAr encoded domain impedes translation of the EBNA1 mRNA [6], [15], [16], [17], [18], [19], [20], the mechanism causing this has remained unclear. There are reports that the EBNA1 GAr polypeptide sequence delays the initiation of EBNA1 mRNA translation [15], [21]. However, other studies have clearly demonstrated that the purine-rich, GAr mRNA structure limits EBNA1 synthesis, resulting in decreased presentation of EBNA1 to specific CD8^+^ T cells [19], [22]. Indeed, recent studies from our group have revealed that the GAr mRNA includes *cis*-regulatory, G-quadruplex structures that inhibit EBNA1 synthesis and thereby modulate the endogenous presentation of EBNA1-specific CD8^+^ T cell epitopes [23].

Considering the importance of CD8^+^ T cells in controlling primary and latent EBV infection, we hypothesized that the translational efficiency of the EBNA1 mRNA may also influence the priming of antigen-specific T cells *in vivo*. To test this hypothesis we used two sequences of the *EBNA1* gene encoding identical proteins but with differential rates of translation of their respective mRNAs to assess the impact of translational efficiency on the induction of effector and memory CD8^+^ T cell responses. A native EBNA1 GAr mRNA inhibits translation due to the presence of G-quadruplex structures, whilst a codon-modified EBNA1 GAr mRNA enhances translation due to destabilization of the G-quadruplex structures [23]. These studies demonstrated that the translational efficiency of the EBNA1 mRNAs directly correlated with the MHC class I antigen presentation *in vivo* and early priming of antigen-specific effector CD8^+^ T cells, while the generation of a memory T cell response was not impacted. Furthermore, the translational efficiency of EBNA1 mRNAs also impacted on the functional profile of antigen-specific effector CD8^+^ T cells, suggesting that changes in their activation are likely related to the amount of antigen available.

## Results

### *Ex vivo* antigen presentation by CD11c^+^ DCs is influenced by mRNA translational efficiency

**Figure 1:**
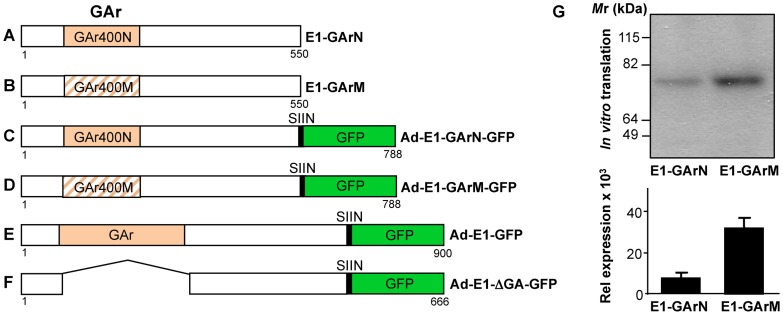
Schematic description of EBNA1 expression constructs comprising different GAr mRNA sequences whilst encoding identical protein sequences. Plasmids expressing EBNA1 were generated in either pcDNA3 for *in vitro* translation studies (A–B) or in Ad5-adenoviral vectors (C–F) for immunological studies. (A–B) EBNA1 encoding 400 nucleotides of either native GAr (E1-GArN) or codon-modified GAr (E1-GArM) respectively; (C–F) EBNA1 encoding 400 nucleotides of either native GAr (Ad-E1-GArN) or 400 nucleotides of codon-modified GAr (Ad-E1-GArM); full-length EBNA1 (Ad-E1) or EBNA1 with a deleted GAr (Ad-E1-ΔGA) were fused in-frame to a C-terminal Green Fluorescent Protein (GFP) to generate (Ad-E1-GArN-GFP); (Ad-E1-GArM-GFP); (Ad-E1-GFP) or (Ad-E1-ΔGA-GFP) respectively. The Ad-EBNA1-GFP expression constructs (C–F) included insertion of a H2K^b^-restricted ovalbumin CTL epitope, SIINFEKL, fused to the EBNA1 C-terminal sequence, allowing analysis of endogenous processing of EBNA1 using a B3Z T-cell hybridoma in the immunological assays as outlined in the Materials and Methods. (G) *In vitro* translation assay of pcDNA3 expression constructs encoding EBNA1 comprising either a native GAr (E1-GArN) or a codon-modified GAr (E1-GArM). Band intensities from the IVT assays were quantified by densitometric analysis using ImageJ64 software. Mean ± SD shown (n = 4).

**Figure 2:**
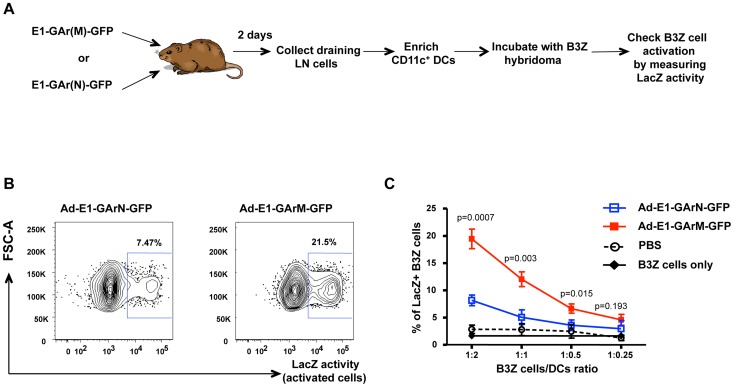
*Ex vivo* antigen presentation by DCs is influenced by mRNA translation efficiency. (A) Flow chart illustrating the experimental procedure. Female C57BL/6 mice were intramuscularly immunized with Ad-E1-GArN-GFP or Ad-E1-GArM-GFP. DLNs were pooled from 8 mice on day 2 following infection. Pan-DCs enriched from DLNs were incubated with B3Z cells at varying effector to target (E∶T) ratios to assess antigen presentation as described in the Materials and Methods. (B) Representative FACS plots showing β-galactosidase activity in B3Z hybridoma incubated with DCs from mice immunized with Ad-E1-GArN-GFP or Ad-E1-GArM-GFP at an E∶T ratio of 1∶2. (C) Overall activation of B3Z cells at varying B3Z cells/DCs ratios from mice immunized with Ad-E1-GArN-GFP or Ad-E1-GArM-GFP.

To determine the impact of EBNA1 mRNA translational efficiency on MHC class I-restricted antigen presentation *in vivo*, we constructed both EBNA1-pcDNA3 (Fig. 1A–B) and adenoviral EBNA1-GFP expression vectors (Fig. 1C–D) to generate mRNAs with differential translational efficiency but encoding identical proteins. These EBNA1 vectors were designed to express either 400 nucleotides of a native GAr mRNA (E1-GArN and Ad-E1-GArN) or 400 nucleotides of a codon-modified GAr mRNA (E1-GArM and Ad-E1-GArM). In addition, we also generated two additional adenoviral EBNA1-GFP expression vectors encoding full-length EBNA1 (Ad-E1-GFP; Fig. 1E) or EBNA1 with a deleted GAr domain (Ad-E1-ΔGAr-GFP; Fig. 1F). The above EBNA1 adenoviral expression vectors all demonstrated similar adenovirus transduction efficiencies (Table S1). To assess the endogenous loading of MHC class I molecules, the adenoviral EBNA1-GFP expression constructs also encoded a model H-2k^b^- restricted CD8^+^ T cell epitope SIINFEKL, comprising OVA_257–264_ from the ovalbumin protein. Recent studies by our group have demonstrated that the native purine-rich EBNA1 GAr mRNA contains G-quadruplexes and these structures have been shown to inhibit EBNA1 self-synthesis [23]. Following codon-modification to reduce this purine-bias we demonstrated that destabilization of the RNA G-quadruplex structures resulted in enhanced EBNA1 synthesis [19], [22] (Fig. 1G). Initially, we challenged C57BL/6 mice with Ad-E1-GArN-GFP or Ad-E1-GArM-GFP. Two days post-infection, CD11c^+^ DCs were enriched from draining inguinal lymph nodes and incubated with B3Z T cells, a CD8^+^ T cell hybridoma cell line that expresses lacZ upon activation of its SIINFEKL-specific T cell receptor (Fig. 2A). CD11c^+^ DCs isolated from mice infected with Ad-E1-GArM-GFP, the more efficiently translated EBNA1 mRNA, demonstrated a significantly higher level of *ex vivo* presentation of the H-2Kb-restricted SIINFEKL epitope (Fig. 2,B–C), which remained consistent at different effector to target ratios (Fig. 2C). These observations indicated that the translation efficiency of viral mRNAs could influence the *in vivo* presentation of CD8^+^ T cell epitopes by professional antigen presenting cells.

### The translational efficiency of homologous viral mRNAs *in vivo* influence DC-T cell interactions and antigen-specific T cell recruitment

**Figure 3:**
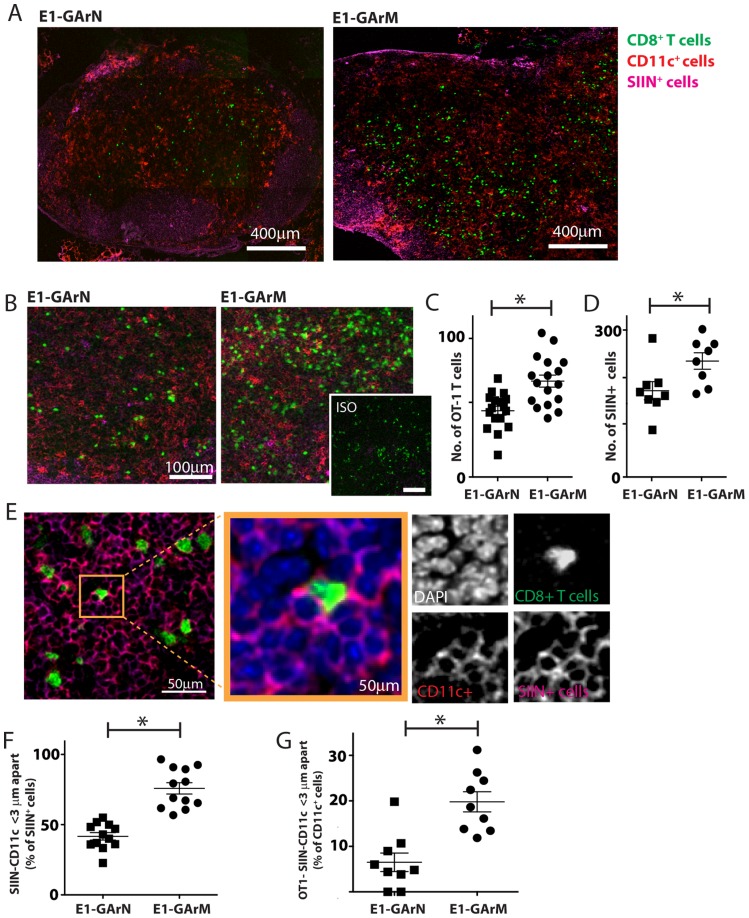
Antigen translation efficiency regulates Ag-specific T cell trafficking and DC-T cell interactions. Female C57BL/6 mice were adoptively transferred with CFSE^+^CD8^+^ OT-1 cells, followed by intramuscular immunization with adenoviral expression vectors Ad-E1-GArN-GFP or Ad-E1-GArM-GFP. DLNs were collected 2 days post-infection for frozen section. These were stained for nucleus, CD11c and Kb-SIINFEKL complex. (A) Representative merged images (×40) show the transferred CFSE^+^CD8**^+^** OT-1 cells, CD11c-expressing cells and SIINFEKL epitope presenting cells. (B) Representative merged images at high magnification (×100) show the transferred CFSE^+^CD8**^+^** OT-1 cells, CD11c-expressing cells and SIINFEKL epitope presenting cells. (C) Comparative analysis of the number of CFSE^+^CD8**^+^** OT-1 cells in DLNs following infection with Ad-E1-GArN-GFP or Ad-E1-GArM-GFP. (D) Comparative analysis of the number of and SIINFEKL epitope presenting cells in DLNs following infection with Ad-E1-GArN-GFP or Ad-E1-GArM-GFP. (E) Representative images show the interactions between CD11c cells and SIINFEKL epitope presenting cells; CFSE**^+^**CD8**^+^** OT-1 cells and SIINFEKL epitope presenting cells. (F) Comparative analysis of the interaction between CD11c cells with H-2Kb-SIIN**^+^** cells. (G) Comparative analysis of the interaction between of H-2Kb-SIIN^+^ CD11c cells with CD8^+^ OT1 effector cells. (*p<0.05, Mann-Whitney).

To further delineate the potential impact of viral mRNA translational efficiency on the modulation of CD8^+^ T cell immunity *in vivo*, we used confocal imaging of draining lymph nodes to visualize the interaction of antigen presenting cells with SIINFEKL-specific CD8^+^ T cells and the recruitment of these effector cells. CFSE-labeled CD8^+^ OT-1 T cells were adoptively transferred into C57BL/6 mice two hours prior to infection with adenoviral expression vectors Ad-E1-GArN-GFP encoding native EBNA1, or Ad-E1-GArM-GFP encoding codon-modified EBNA1. Draining lymph nodes were harvested on day 2 for frozen section and stained with anti-CD11c, anti-H-2Kb-SIIN (25-D1.16) and DAPI. Mice infected with the vector encoding native EBNA1, demonstrated a significantly reduced number of CFSE^+^CD8^+^ OT-1 cells and H-2Kb-SIIN^+^ APCs in draining lymph nodes when compared to mice infected with the vector encoding codon-modified EBNA1 which generates a faster translating EBNA1 mRNA due to destabilization of the native G-quadruplex structures (Fig. 3A–D). Additionally, we observed that mice infected with the vector expressing native EBNA1 also demonstrated a significantly lower number of H-2Kb-SIIN^+^ APCs within 3 µm distance of CFSE^+^CD8**^+^** OT-1 cells compared to mice challenged with the vector expressing the codon-modified EBNA1, suggesting enhanced interactions between APC and CD8^+^ T cells in mice immunized with a vector encoding a codon-modified EBNA1 (Fig. 3E–G). These observations further emphasize the critical role of translational efficiency of viral mRNAs in not only modulating antigen presentation *in vivo* but also influencing the number of effector T cells and in the interaction of APCs and antigen-specific T cells in draining lymph nodes.

### Differential translation of homologous EBNA1 mRNAs impacts on CD8^+^ effector T cell proliferation, transcriptional and polyfunctional profiles

**Figure 4:**
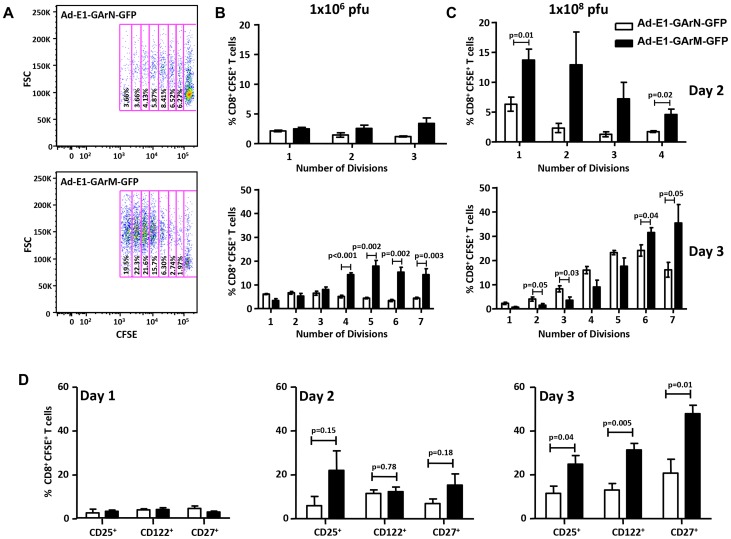
T cell proliferation and activation are influenced by differentially translated EBNA1 mRNAs. (A) Representative FACS plot shows the proliferation of transferred CD8**^+^** OT-1 cells in murine DLNs 3 days post-infection with Ad-E1-GArN-GFP or Ad-E1-GArM-GFP. The numbers in each box represent the percentage of CFSE labeled CD8**^+^** OT-1 cells in murine DLNs. Each box represents one round of cell division. FSC: forward scatter. (B–C) Data represent the mean ± S.E.M. of the percentage of OT-1 CD8^+^ T cells in each division on days 2 and 3 post-infection with Ad-E1-GArN-GFP or Ad-E1-GArM-GFP at two different viral dosages (1×10^6^ pfu/mouse) or (1×10^8^ pfu/mouse). (D) Expression of CD25, CD122 and CD27 on days 1, 2 and 3 by transferred CD8^+^ OT-1 cells following infection with Ad-E1-GArN-GFP or Ad-E1-GArM-GFP (1×10^6^ pfu/mouse). Each vertical bar represents the mean ± SD (n = 5).

Having established a direct impact of mRNA translational efficiency on *in vivo* antigen presentation, APC-T cell interactions and antigen-specific T cell numbers in draining lymph nodes, we next assessed the impact of differential translation efficiency on antigen-specific CD8^+^ T cell proliferation and activation. C57BL/6 mice were adoptively transferred with CFSE-labelled CD8^+^ OT-1 cells (5×10^6^ cells/mouse) and subsequently challenged with Ad-E1-GArN-GFP or Ad-E1-GArM-GFP (1×10^6^ or 1×10^8^ pfu/mouse). Mice were sacrificed on days 2 and 3 post-infection and the proliferation, activation and functional properties of the adoptively transferred OT-1 cells were evaluated from draining inguinal lymph nodes. Proliferation of OT-1 cells was significantly lower in mice challenged with an adenoviral vector encoding native EBNA1, Ad-E1-GArN-GFP, (Fig. 4A–C). Interestingly, the kinetics of proliferation of these effector cells correlated with the dose of viral infection. We confirmed these results by infection with a full-length native EBNA1-GFP adenoviral expression construct, Ad-E1-GFP, which also showed low T cell proliferation, whilst infection with an adenoviral EBNA1-GFP expression vector where the GAr was deleted, Ad-E1-ΔGA-GFP, resulted in enhanced proliferation of antigen-specific CD8^+^ T cells (data not shown).

As the (IL-2/IL-2R) pathway is crucial for T-cell activation, proliferation and differentiation [24], [25] we next assessed the expression of IL-2 receptors on CD8^+^ T cells. Antigen-specific CD8^+^ T cells from mice challenged with an adenoviral vector encoding codon-modified EBNA1, Ad-E1-GArM-GFP, led to a significantly higher proportion of these cells being CD25^+^ (IL-2Rα) and CD122^+^ (IL-2Rβ) (Fig. 4D). In addition, as CD27 plays a pivotal role in the generation, maintenance and differentiation of cytotoxic T lymphocytes [26], [27], the expression of CD27 was also assessed and we observed that the majority of SIINFEKL-specific CD8^+^ T cells isolated from mice challenged with an adenoviral vector encoding codon-modified EBNA1, Ad-E1-GArM-GFP, were also CD27^+^ (Fig. 4D), suggesting that T cells primed with this vector are less differentiated and are more likely to respond to IL-2.

**Figure 5:**
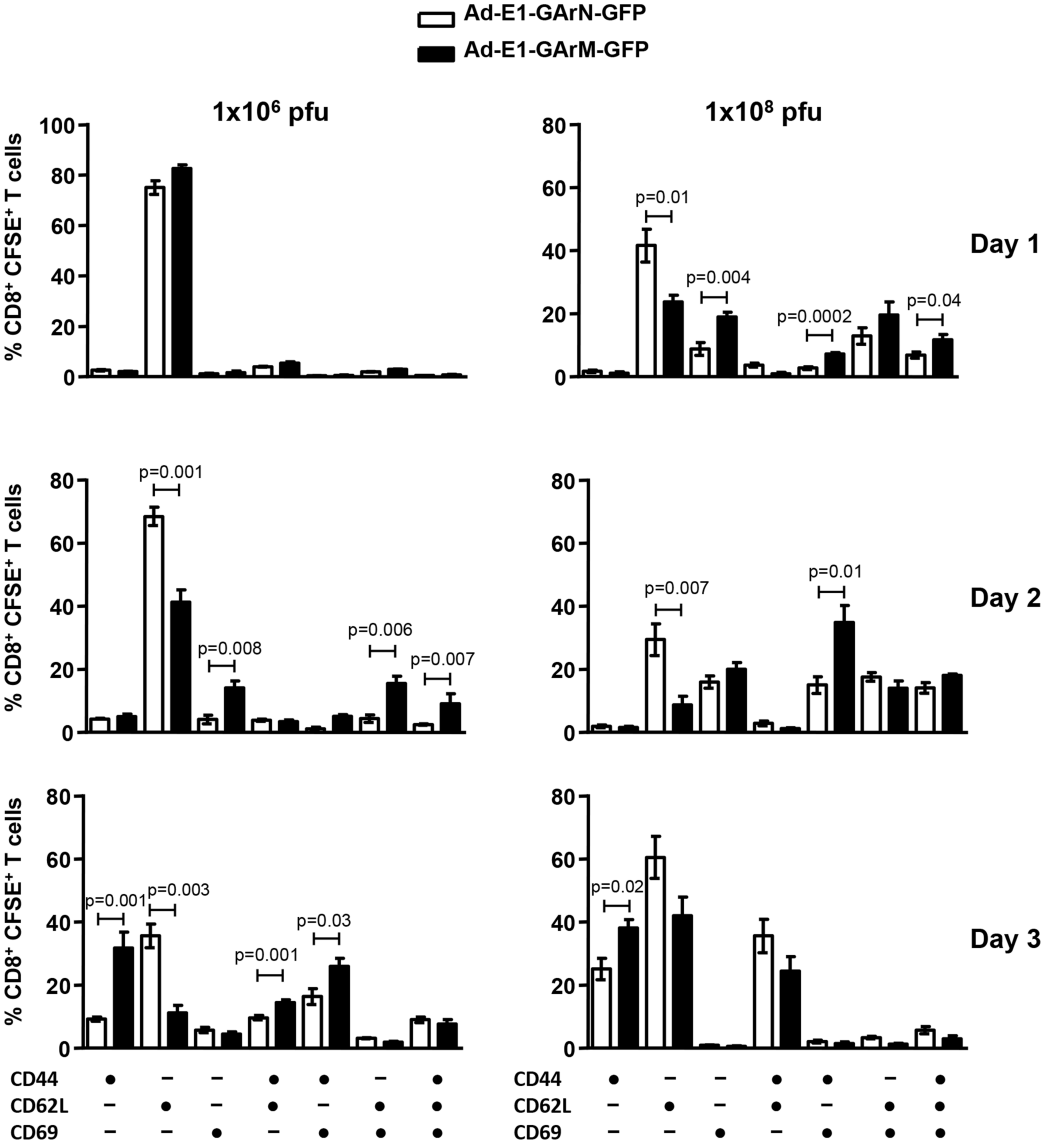
T cell activation is influenced by differentially translated EBNA1 mRNAs. Expression of the T cell activation markers CD44, CD62L and CD69 in the DLNs of mice infected with Ad-E1-GArN-GFP or Ad-E1-GArM-GFP (1×10^6^ pfu/mouse or 1×10^8^ pfu/mouse) on days 1, 2 and 3. Each vertical bar represents the mean ± S.E.M. (n = 5).

Consistent with the data presented above, we also observed a significant difference in the activation profile of SIINFEKL-specific CD8^+^ T cells. Mice infected with an adenoviral vector encoding EBNA1 with a codon-modified GAr domain, Ad-E1-GArM-GFP, demonstrated a significantly higher proportion of activated antigen-specific CD8^+^ T cells (Fig. 5). These T cells included CD44^+^CD62L^−^CD69^−^ and CD44^+^CD62L^−^CD69^+^ populations. Interestingly, mice infected with an adenoviral vector encoding EBNA1 with a native GAr domain, Ad-E1-GArN-GFP, showed a significantly higher number of CD44^−^CD62L^+^CD69^−^ antigen specific T cells in the DLNs, further emphasizing the impact of G-quadruplex structures on the activation of T cells *in vivo* (Fig. 5). It should be noted that the kinetics of T cell activation correlated with the dose of viral infection. Infection with high doses of adenoviral vectors (1×10^8^ pfu/mouse) was co-incident with early activation of antigen-specific T cells (day 2), while the peak of activation in mice challenged with a lower dose of virus (1×10^6^ pfu/mouse) was not observed until day 3 (Fig. 5).

**Figure 6:**
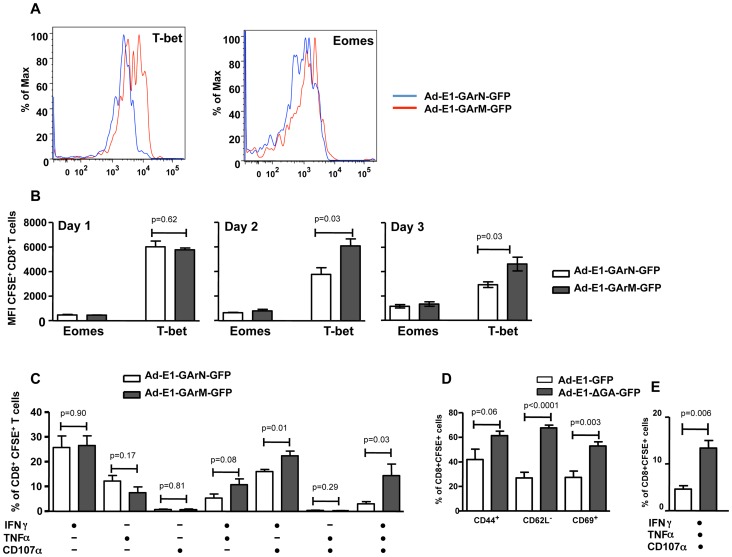
Differential translation of EBNA1 mRNAs impacts on the expression of transcriptional factors and antigen specific functions in Ag-specific T cells from DLNs. Female C57BL/6 mice were adoptively transferred with CFSE^+^CD8^+^ OT-1 cells, followed by intramuscular immunization with Ad-E1-GArN-GFP or Ad-E1-GArM-GFP virus (10^6^ pfu/mouse) 2 hours following transfer. Mice were sacrificed on days 1, 2 or 3 post-infection. Expression of T-bet and Eomes in transferred CD8**^+^** OT-1 cells was evaluated. (A) Representative FACS plots show the expression of T-bet and Eomes in CFSE^+^CD8**^+^** OT-1 cells. (B) Overall results of the expression of T-bet and Eomes in CFSE^+^CD8**^+^** OT-1 cells at different time points post-infection with Ad-E1-GArN-GFP or Ad-E1-GArM-GFP. (C) DLN cells were prepared from mice on day 3 post-infection with Ad-E1-GArN-GFP or Ad-E1-GArM-GFP and the expression of IFN-γ, TNF-α and translocation of CD107α by transferred CD8**^+^** OT-1 cells was evaluated using a standard 6 hour ICS assay. (D–E) Female C57BL/6 mice were adoptively transferred with CFSE-labeled CD8^+^ OT-1 cells (1×10^6^ cells/mouse) and immunized intramuscularly with Ad-E1-GFP or Ad-E1-ΔGA-GFP virus (1×10^6^ pfu/mouse) 2 hours following transfer with DLNs harvested on day 3 post-infection to assess the activation and functions of antigen-specific CD8^+^ T cells. (D) The expression of T cell activation markers CD44, CD62L and CD69 on CFSE^+^CD8**^+^** OT-1 cells (E) Expression of IFN-γ, TNF-α and translocation of CD107α by transferred CD8**^+^** OT-1 cells. Mean ± SD shown (n = 5).

Previous reports have demonstrated that the T-box transcriptional factors, T-bet and Eomesodermin (Eomes), play crucial roles in regulating T cell differentiation and function including expression of cytokines and cytotoxicity [28], [29], [30], [31]. *Ex vivo* analysis of SIINFEKL-specific effector T cells demonstrated that mice infected with Ad-E1-GArM-GFP showed significantly higher levels of T-bet expression on days 2 and 3 post-infection compared to mice infected with Ad-E1-GArN-GFP (Fig. 6A–B). In contrast, the levels of Eomes in antigen-specific effector T cells were not significantly different in mice infected with Ad-E1-GArM-GFP or Ad-E1-GArN-GFP (Fig. 6A–B). To investigate further the potential impact of T-bet expression, we assessed the polyfunctional potentiality of antigen-specific T cells. For these analyses, T cells from draining lymph nodes were stimulated *in vitro* with SIINFEKL peptide and assessed for expression of the anti-viral cytokines IFNγ and TNFα and the cytotoxicity degranulation marker CD107. T cells from the Ad-E1-GArM-GFP-infected mice displayed significantly higher polyfunctional potentiality (IFNγ^+^, TNFα^+^ and CD107α^+^ or IFNγ^+^ and CD107α^+^) when compared to Ad-E1-GArN-GFP-infected mice (Fig. 6C). Similarly, infection with Ad-E1-ΔGA-GFP, where the GAr domain has been deleted resulting in improved translation of the E1-ΔGA-GFP mRNA, also showed significantly enhanced expression of activation markers (Fig. 6D) and polyfunctional antigen-specific T cells (Fig. 6E) compared to infection with Ad-E1-GFP, a construct expressing full-length native EBNA1. Mice infected with a control recombinant EBNA1-GFP expression vector not encoding SIINFEKL showed no antigen-specific T cell responses when stimulated *in vitro* with SIINFEKL peptide (Fig. S1).

**Figure 7:**
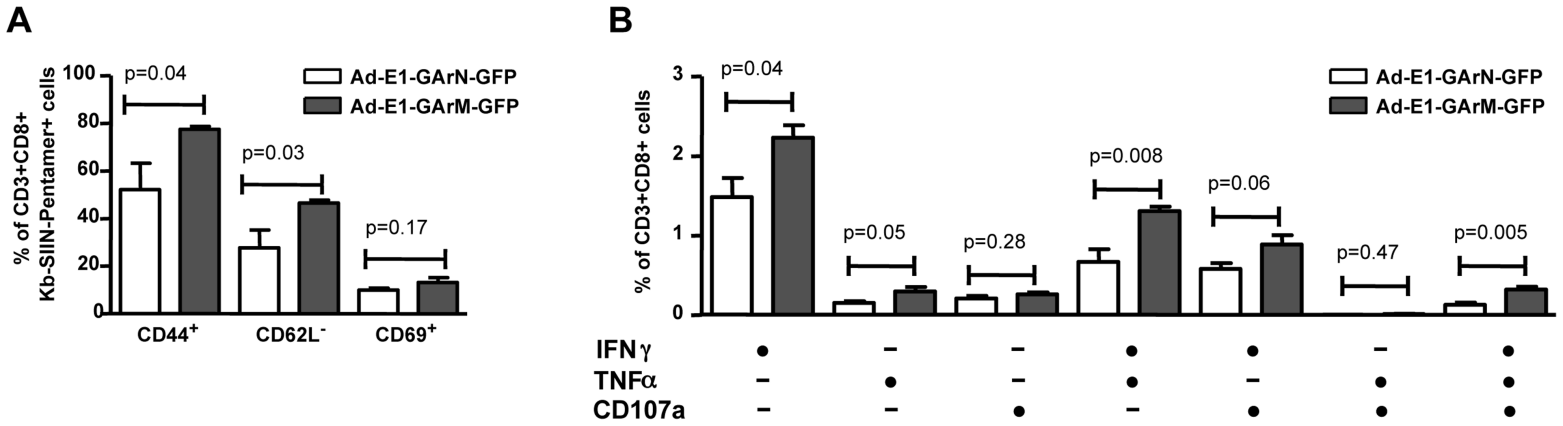
The differential translation of EBNA1 mRNAs impacts on the activation and functional programming of antigen-specific T cells. Female C57BL/6 mice were immunized intramuscularly with Ad-E1-GArN-GFP or Ad-E1-GArM-GFP virus (A–B). Mice were sacrificed on day 7 post-infection and spleen cells harvested to evaluate the activation and functions of antigen-specific CD8^+^ T cells. (A) Expression of T cell activation markers CD44, CD62L and CD69. (B) Expression of IFN-γ, TNF-α and translocation of CD107α by Ag-specific CD8**^+^** OT-1 cells. Mean ± SD shown (n = 5).

To confirm that these results were not due to an artifact of adoptive T cell transfer, we repeated these studies in C57BL/6 mice without cell transfer. Naïve mice were challenged with Ad-E1-GArN-GFP or Ad-E1-GArM-GFP and the expression of early activation markers and polyfunctional potentiality of primary SIINFEKL-specific effector T cells were assessed on day 7 using an H-2K^b^-SIIN pentamer. Similar to the results demonstrated in the adoptive T cell transfer setting, we observed a significantly higher proportion of T cells from naïve C57BL/6 mice infected with Ad-E1-GArM-GFP, which were of the activated phenotype CD44^+^CD69^+^CD62L^−^ and which displayed polyfunctional potentiality compared to the T cells from naïve C57BL/6 mice infected with Ad-E1-GArN-GFP (Fig. 7A–B).

**Figure 8:**
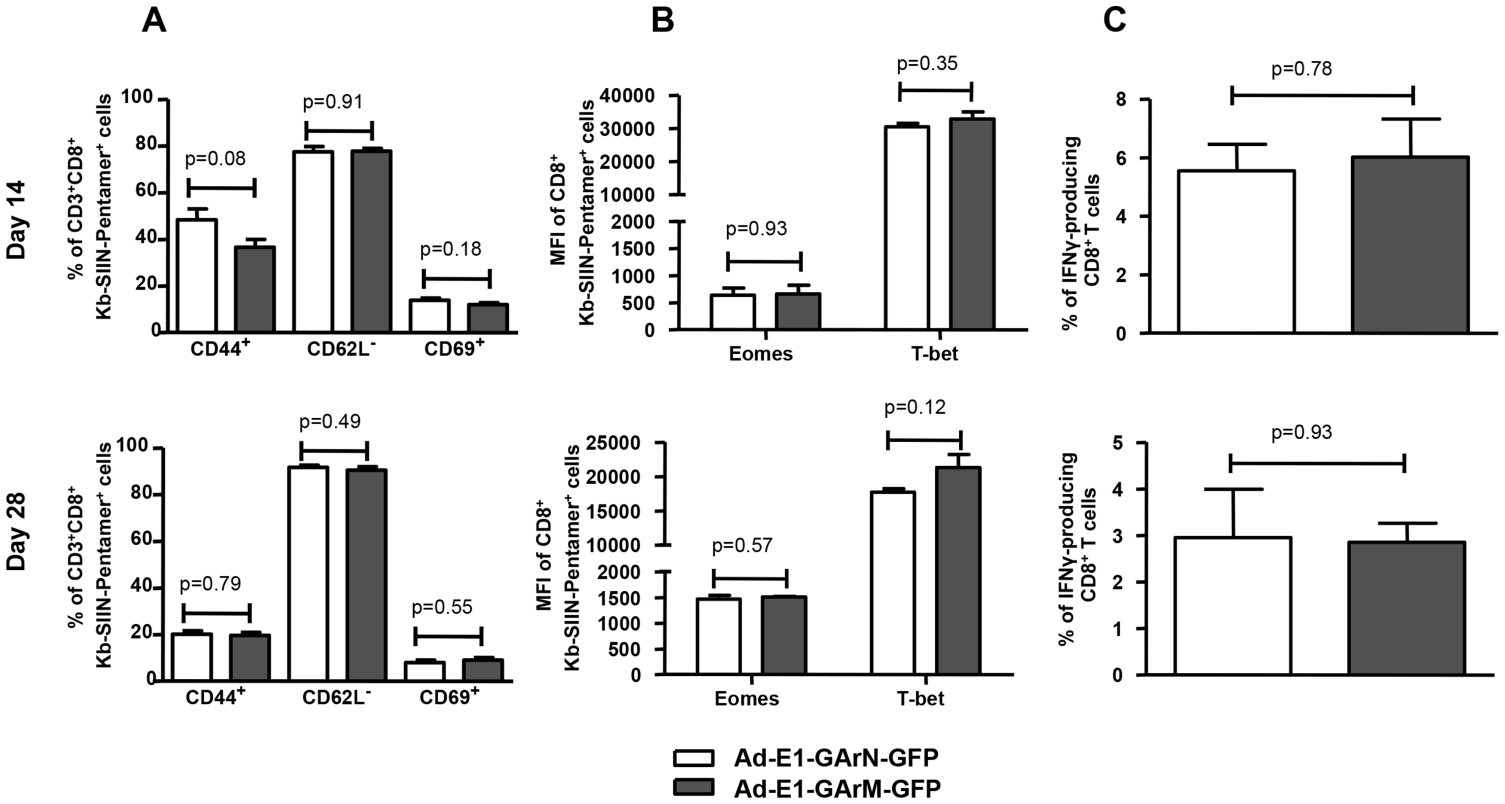
Differentially translated EBNA1 mRNAs have a minimal effect on Antigen-specific memory responses. Female C57BL/6 mice were immunized intramuscularly with Ad-E1-GArN-GFP or Ad-E1-GArM-GFP virus. Spleen cells were collected on day 14 (top panel) or day 28 (lower panel) post-infection. (A) Expression of T cell activation markers of CD44, CD62L and CD69 on SIINFEKL-specific CD8^+^ T cells. (B) Expression of transcriptional factors Eomes and T-bet in SIINFEKL-specific CD8^+^ T cells. (C) The expression of IFN-γ by SIINFEKL-specific CD8**^+^** T cells was evaluated using a standard 6- hour ICS assay. Mean ± SD shown (n = 5).

We also investigated whether the impact of mRNA translational efficiency on an early effector T cell response also extends to the generation and establishment of a memory response. C57BL/6 mice were challenged with Ad-E1-GArN-GFP and Ad-E1-GArM-GFP and SIINFEKL-specific CD8^+^ T cell responses were assessed on days 14 and 28. Antigen-specific T cells from mice infected with Ad-E1-GArN-GFP and Ad-E1-GArM-GFP demonstrated similar levels of activation markers (Fig. 8). Furthermore, these T cells also showed comparable levels of the T-box transcription factors T-bet and Eomes and a similar capacity to express IFNγ following stimulation with SIINFEKL peptide. Taken together, these data clearly demonstrate that the native EBNA1 GAr mRNA comprising G-quadruplex structures limits the availability of peptide/MHC complexes on the cell surface of antigen presenting cells and selectively impacts on the functional quality of an early antigen-specific T cell response *in vivo*.

## Discussion

Although the innate immune response is crucial in controlling early stages of viral infection, rapid priming of an effective adaptive cellular immune system can determine the outcome of primary viral infection [32], [33]. In particular, recruitment of virus-specific CD8^+^ T cells and their maturation as effector cells is achieved through an interaction between T-cell receptors and MHC class I-peptide complexes on professional antigen presenting cells [34]. Following activation, these effector T cells scan the surface of virus-infected cells to detect viral peptides bound to MHC class I molecules and eliminate these cells either by direct lysis or by secreting cytokines/chemokines [35]. To counter this effector mechanism, viruses have evolved multiple strategies to limit the endogenous processing and presentation of viral peptides [36], [37]. Indeed, human herpes viruses including EBV, which are known to establish persistent infection, have developed specific strategies to suppress the expression of immunodominant antigens [38], [39]. While limiting the supply of viral peptides by controlling the synthesis of viral proteins can prevent T cell recognition, this strategy can also impact on viral fitness and compromise the ability of viruses to establish persistent infection [40]. EBV-encoded EBNA1 is a good example of a viral protein, which has successfully evolved unique strategies to overcome this dilemma. This protein includes an internal glycine-alanine repeat domain that not only limits self-synthesis but also blocks proteasomal degradation [6], [13]. While restricted expression of this protein limits the availability of peptide epitopes, the blockade of degradation provides sufficient protein for maintenance of the viral genome in virus-infected B cells.

A number of studies published over the last decade have shown that in spite of the inhibitory effect of the internal GAr domain, CD8^+^ T cell responses directed towards EBNA1 can be readily detected in EBV seropositive individuals [2], [3], [41]. This paradox was resolved following extensive *in vitro* molecular analysis of the endogenous processing of EBNA1 which showed that CD8^+^ T cell epitopes from this protein were predominantly generated from newly synthesized RDPs rather than from the long-lived pool of stable EBNA1 in EBV-infected B cells [2], [3], [6], [15], [17], [19], [21], [22], [40]. These observations have subsequently been further extended to demonstrate that the generation of RDPs is intrinsically linked to the rate at which proteins are synthesized [17]. It has been reported that EBNA1 with its native GAr domain generates RDPs less efficiently compared to EBNA1ΔGA, which is more efficiently synthesized [20]. As a consequence, CD8^+^ T cell epitopes generated from EBNA1ΔGA were more efficiently processed and cells expressing EBNA1ΔGA were lysed *in vitro* at higher levels by EBV-specific CTLs compared to cells expressing a poorly translated full-length EBNA1. Based on these observations, we hypothesized that an EBNA1 GAr-mediated inhibitory effect on mRNA translation and RDP generation may be one of the crucial mechanisms by which EBV modulates the kinetics of antigen presentation and the priming of virus-specific CD8^+^ T cell responses *in vivo*. Indeed, it has been recently revealed that the EBNA1 GAr mRNA exploits G-quadruplex structure to inhibit translation elongation by impeding ribosome transit, thereby down-regulating EBNA1 synthesis and limiting the availability of peptide epitopes.

The identification of inhibitory G-quadruplex structures within EBNA1 has helped elucidate an important EBNA1 translational regulatory mechanism. Earlier studies had proposed that the GAr peptide sequence may interfere with translation initiation to down-regulate EBNA1 synthesis [15], however toe-printing experiments performed by the Shastri group demonstrated that inhibition of EBNA1 synthesis was most likely not due to interference of translation initiation [20]. Results from recent EBNA1 polysome profiling experiments demonstrated that G-quadruplex structural elements within the EBNA1 GAr mRNA act as steric blocks to cause a stalling/dissociation of ribosomes [23]. This result confirmed previous findings that reducing the purine-bias within the EBNA1 GAr mRNA through codon-modification, whilst maintaining the encoded protein sequence results in increased EBNA1 mRNA translation [22], [23]. We have more recently demonstrated that codon-modification of the repeat sequence leads to destabilization of the G-quadruplex structures within the GAr, which in turn leads to increased EBNA1 mRNA translation. In the present study, EBNA1 variants displaying distinct translational efficiencies for their respective native or codon-modified mRNAs have been used to assess the influence of mRNA translational efficiency on both *in vivo* antigen presentation and priming of virus-specific CD8^+^ T cell responses. Comparison of the *ex vivo* antigen presentation by CD11c^+^ DCs from the DLNs of mice infected with adenoviral vectors encoding E1-GArN-GFP or E1-GArM-GFP revealed that CD8^+^ T cell epitopes from these proteins were differentially presented on the cell surface and this presentation correlated with their translational efficiency. These observations extend previously published data on the impact of translational efficiency on T cell recognition of EBNA1 expressing virus-infected cells *in vitro*. While EBNA1 mRNA in humans is primarily expressed in B cells *in vivo*, previous studies have suggested that dendritic cells may play an important role in the priming of naïve T cells, which recognize EBV latent antigens [42], [43]. This dendritic cell-mediated priming may be mediated through either direct infection of these cells with EBV [44], [45], [46] or through cross-presentation [47].

Having established that mRNA translational efficiency is intrinsically linked to *in vivo* antigen presentation by professional APCs, we further demonstrated the impact of mRNA translational efficiency on CD8^+^ T cell priming by *ex vivo* imaging of draining lymph nodes which showed enhanced antigen-specific T cell-APC interactions in mice infected with an adenoviral vector expressing a rapidly translated EBNA1 mRNA (Ad-E1-GArM-GFP). More importantly, these antigen-specific T cells displayed superior polyfunctionality and increased expression of the T-box transcription factor, T-bet. Earlier studies, primarily in animal models, have demonstrated the critical role of T-box transcription factors in regulating effector function and the establishment of CD8^+^ T cell memory. Thus antigen-specific T cells with high T-bet expression display long-term resilience and protection from CD8^+^ T cell exhaustion. Recent studies have demonstrated that the level of T-bet expression in virus-specific CD8^+^ T cells is associated with the efficiency of endogenous antigen presentation, clonal expansion and the phenotypic and functional profiles of antigen-specific T cells [48]. Data presented in this study further extend these observations and provide evidence for a potential link between mRNA translational efficiency and antigen presentation *in vivo* and its impact on the functional programming of effector T cells. We note that in spite of a dramatic impact of mRNA translational efficiency on early priming of CD8^+^ T cells, we observed only a minimal impact on the generation of memory CD8^+^ T cell responses.

The above observations provide an important insight into how persistent viruses like EBV exploit mRNA translational efficiency to modulate antigen presentation *in vivo*. In such a setting, viruses would restrict early priming of antigen-specific T cells, thereby escaping immune surveillance and allowing the establishment of a successful latent infection. Furthermore, the techniques utilized here may provide a general method to improve the immunogenicity of poorly immunogenic viral proteins which restrict T cell priming by limiting the availability of epitopes on the surface of virus-infected cells. Enhanced mRNA translation through codon-modification can dramatically enhance the endogenous presentation of immunodominant epitopes by professional APCs and induce a strong effector T cell response. We predict that these effector cells will recognize virus-infected cells more efficiently compared to naturally-induced anti-viral T cells. These findings also suggest a novel and alternate platform for designing anti-viral strategies that focus on targeting RNA structure rather than protein within gammaherpesviral ORFs. Such strategies may include antisense oligonucleotides to enhance viral protein synthesis and the early priming of effector T cells, to establish a more rapid immune response and prevent persistent infection.

## Materials and Methods

### Ethics statement

This study was performed under strict accordance with the recommendations in the Guide for the Care and Use of Laboratory Animals of the National Health and Medical Research Council (Australia). The Animal Ethics Committee of the QIMR Berghofer Medical Research Institute approved the protocol [Protocol approval number: P353 (A0610-612)]. All viral infections were performed under gaseous (isoflurane) anaesthesia and every effort was made to minimize suffering.

### Cell lines

HEK293 cells were cultured in Dulbecco modified Eagle medium supplemented with 10% fetal bovine serum, 2 mM Glutamax, and 100 IU/ml penicillin-streptomycin. The SIINFEKL-specific CD8^+^ T-cell hybridoma (B3Z) was maintained in RPMI 1640 supplemented with 2 mM L-glutamine (Gibco, Life Technology), 1 mM sodium pyruvate (Gibco, Life Technology), 50 µM 2-mercaptoethanol (Gibco, Life Technology), 100 IU/ml penicillin, and 100 µg/ml streptomycin plus 10% FCS (R-10).

### Antibodies and pentamer

APC-anti-CD3e, PE-Cy7-anti-CD3e, FITC-anti-CD3e, PE-anti-CD8a, PerCp-anti-CD8a, Alex700-anti-CD19, FITC-anti-CD44, APC-anti-CD44, V450-anti-CD44, PE-anti-IFN-γ, PE-Cy7-anti-TNF-α were purchased from BD Biosciences (Australia); PE-Cy7-anti-CD25, FITC-anti-CD27, APC-anti-CD27, Alexa647-anti-CD107a, eFluo450-anti-CD122, PE-anti-T-bet and Alexa700-anti-Eomes and purified anti-mouse MHC I-SIINFEKL complex (clone 25D) were purchased from eBioscience (Jomar Bioscience, Kensington, SA., Australia); PerCp-Cy5.5-anti-CD62L, PE-Cy7-anti-CD69 and purified anti-CD11c were purchased from BioLegend, (Australian Biosearch, Karrinyup, WA., Australia); APC-H-2Kb-SIINFEKL pentamer was purchased from ProImmune (Oxford, UK).

### Mice

C57BL/6 mice were purchased from Animal Resource Centre (Perth, WA, Australia). OT-1 mice were bred and maintained under conventional conditions in the animal facility at the Queensland Institute of Medical Research. The Queensland Institute of Medical Research Animal Ethics Committee approved and monitored all animal procedures.

### Generation of recombinant adenovirus encoding EBV EBNA1

Full-length EBV-encoded EBNA1 (E1), EBNA1 with a deleted GA repeat (E1-ΔGAr), EBNA1 expressing 400 nucleotides of native GAr (E1-GAr400N) referred to as E1-(GArN) and EBNA1 expressing 400 nucleotides of codon-modified GAr (E1-GAr400M) referred to as E1-(GArM) had been previously subcloned in-frame with a sequence coding for GFP (pEGFP-N1; Clontech) to generate E1-GFP, E1-ΔGAr-GFP, E1-GArN-GFP and E1-GArM-GFP, respectively [2], [17]. To enable the analysis of endogenous processing of these proteins, a sequence encoding a previously defined H-2K^b^–restricted epitope SIINFEKL (referred to as SIIN) was inserted into the EBNA1-GFP expression constructs between the 3′ end of the EBNA1 sequence and the start of the GFP sequence. Recombinant adenovirus encoding E1-SIIN-GFP, E1-ΔGAr-SIIN-GFP, E1-GArN-SIIN-GFP and E1-GArM-SIIN-GFP were generated by a highly efficient, ligation-based protocol of Adeno-X System (Clontech) [2], [49]. Briefly, E1-GArN-SIIN-GFP was cloned into the pshuttle plasmid between *Nhe*I and *Xba*I restriction enzyme sites and transformed with dam negative cells (SCS110). E1-GArM-SIIN-GFP, E1-ΔGAr-SIIN-GFP and E1-SIIN-GFP were all digested with *BspE*1 and *Xcm*I enzymes and cloned into E1-GArN-SIIN-GFP-pshuttle 2 (replacing the 400 nucleotides of native GAr sequence with either the full-length GAr, no GAr or 400 nucleotides of codon-modified GAr) to generate Ad-E1-SIIN-GFP, Ad-E1-ΔGAr-SIIN-GFP, Ad-E1-GArN-SIIN-GFP and E1-GArM-SIIN-GFP, respectively. Viral titers were verified using an end-point dilution assay. The viral infection efficiency of all constructs was assessed with varying dilutions into HEK293 cells and by measuring GFP fluorescence using flow cytometry.

### *In vitro* translation assays

EBNA1-pcDNA3 constructs expressing 400 nucleotides of either native or codon-modified GAr (E1-GArN; E1-GArM) were transcribed and translated *in vitro* with T7 RNA polymerase using a coupled transcription/translation reticulocyte lysate system (Promega, Madison WI) supplemented with 10 µCi ^35^[S]-methionine (Perkin-Elmer Pty Ltd., Boston, MA.). Lysates were subjected to SDS-PAGE followed by autoradiography and band intensities were quantified by densitometric analysis using ImageJ64 software.

### B3Z T-cell activation assay

B3Z is a T-cell hybridoma expressing a TCR that is specifically activated by the Ovalbumin (257–264) peptide, (SIINFEKL), in the context of H-2Kb. The cells express the beta-galactosidase (lacZ) gene under the control of the nuclear factor of activated T-cell (NF-AT) element of the interleukin 2 enhancer, thereby allowing the activation of B3Z T-cells to be measured by β-galactosidase activity in single cells [50]. SIINFEKL-expressing EBNA1 transfectants were incubated with B3Z cells at varying effector to target cell ratios for 18 h. B3Z cells were harvested, stained with anti-mouse CD3 and anti-mouse CD8 antibodies, followed by osmotic loading of FDG (fluorescein di-β-galactoside; Invitrogen), as previously described [51]. Briefly, B3Z cells were resuspended in 25 µl staining buffer (PBS containing 4% FBS and 10 mM HEPES, pH 7.2), incubated for 10 minutes at 37°C followed by the addition of 25 µl pre-warmed FDG (2 mM in de-ionized water). After a further incubation at 37°C for 1 minute, 450 µl ice-cold staining buffer containing 1 µg/ml 7-Aminoactino-mycin D (7-AAD) was added to each sample to stop osmotic loading. Cells were kept on ice until assessment by flow cytometry on a FACSCanto (BD Biosciences). Live B3Z cells (7-AAD^−^CD3^+^CD8^+^ cells) were gated to analyze their activation by measuring intracellular β-galactosidase activity.

### Detection of antigen presentation by DCs

SIINFEKL-specific B3Z hybridoma cells were used to detect cells presenting SIINFEKL peptide as previously described [50], [52]. Briefly, C57BL/6 mice were immunized with either E1-GArM-GFP or E1-GArN-GFP at 1×10^8^ pfu/mouse. DLNs were collected 2 days post-infection and DCs enriched from DLNs of immunized mice and incubated with B3Z cells at different ratios overnight in R-10. The cells were then harvested, loaded with FDG and analyzed for LacZ activity on a FACSCanto.

### Confocal microscopy

CD8^+^ T cells were enriched from the spleens of OT-1 mice using a mouse CD8^+^ T cell isolation kit II (Miltenyl Biotec Australia). The purified CD8^+^ OT-1 cells were labeled with 0.5 µM carboxyfluorescein succinimidyl ester, CFSE, (Sigma, Mo. USA) and adoptively transferred intravenously into C57BL/6 mice (5×10^6^ cells/mouse). Two hours following adoptive transfer, the mice were immunized intramuscularly with either Ad-E1-GArN-SIIN-GFP or Ad-E1-GArM-SIIN-GFP at a dose of 1×10^8^ pfu/mouse (3 mice/group). Draining inguinal lymph nodes were removed 48 hours later and snap frozen in an optimal cutting temperature (OCT) buffer (ProSci Tech, Qld. Australia). Frozen sections (7 µm) were fixed in 75% acetone 25% ethanol for 5 minutes, washed in PBS and blocked with Vector Biotin Blocking kit for 2×10 minutes, followed by 60 min block in Biocare Medical Mouse Block plus donkey anti-mouse Fab fragments. Sections were washed in PBS before a further block in 10% normal donkey serum in PBS for 30 minutes. Slides were stained with anti-CD11c and anti-H2Kb SIIN or isotype control antibodies for 60 min at room temperature followed by biotinylated goat anti-Armenian hamster for 30 min. MACH1 Mouse Probe was applied for 15 minutes. Sections were stained with Alexa donkey anti-rabbit 647, streptavidin-555 applied for 30 minutes and incubated with DAPI for 10 minutes before mounting with Dako Fluorescent mounting media. Slides were examined on a Zeiss 512 Meta confocal microsope with a ×25 oil emersion objective lens, taking 2 µm Z-sections, or ×60 objective with 2 µm Z-sections. Tiling was used for whole-section imaging. Images for publication were cropped, minimally adjusted for brightness and contrast, and a 3×3 median filter applied using ImageJ and Photoshop. Raw images were analyzed using Imaris 7.6.4: determining baseline fluorescence parameters from controls, identifying cell types by fluorescence and size, and using spot-analysis to calculate XYZ intercellular distances. Parameters were saved and applied to all images within an experiment to maintain objective consistency. At least 3 fields of view per slide of each mouse were used for data analysis. Experiments were repeated at least twice. Data were exported into Excel and Graphpad Prism for statistical analysis. One-way ANOVA was applied to compare groups, which we considered statistically significant if p<0.05.

### Adoptive transfer of CD8^+^ OT-1 T cells

CD8^+^ OT-1 T cells were purified from the spleens of OT-1 mice using a mouse CD8a^+^ T cell isolation kit II (Miltenyi Biotec Australia, Cat# 130-095-236) according to the manufacturer's instruction. The isolated CD8^+^ OT-1 T cells were then labeled with 5 µM CFSE as previously described [53] and adoptively transferred to female C57BL/6 mice by intravenous injection. Recipient mice were immunized with recombinant adenovirus two hours following adoptive transfer.

### Intracellular cytokine staining

CD8^+^ OT-1 T cells were surface stained with APC-H-2Kb-SIINFEKL pentamer for 20 minutes then with anti-CD8a, anti-CD19 before fixation and permeabilization and intracellular staining for IFN-γ and TNF-α. CFSE^+^CD8^+^ OT-1 T cells were first surface stained with anti-CD8a and anti-CD3e mAbs, fixed and permeabilized using cytofix/cytoperm (BD Biosciences, San Diego, CA) and then labeled with anti-IFN-γ and anti-TNF-α mAbs. Pluripotent CTL responses were evaluated by ICS as previously described [54]. Briefly splenocytes or local draining lymph node (DLN) cells were restimulated with SIINFEKL peptide (0.1 µg/ml) for 6 hours at 37°C in complete DMEM (high glucose, Invitrogen, Grand Island, NY, USA) medium supplemented with 10% FCS, 1 mM sodium pyruvate (Gibco, Life Technology), MEM non-essential amino acids (Gibco, Life Technology), 50 µM 2-mercaptoethanol, 100 U/ml penicillin and 100 U/ml streptomycin (D-10). Anti-CD107a antibody was added to the culture at the beginning. Brefeldin A and monensin (BD Biosciences, San Diego, CA) were added 1 hour later to prevent cytokine release. Cells were harvested after 6 hours for staining.

### T cell activation marker and transcriptional factor staining

To determine the activation status of T cells after adoptive transfer, splenocytes and local DLN cells were stained with anti-CD8a, anti-CD44, anti-CD62L, anti-CD69, anti-CD25, anti-CD27 and anti-CD122. In other experiments splenocytes and local DLN cells were stained with APC-H-2Kb-SIINFEKL pentamer for 20 minutes, followed by staining with anti-CD8a, anti-CD19, anti-CD44, anti-CD62L, anti-CD69, or anti-CD25, anti-CD27 and anti-CD122. Transcriptional factor expression was determined by intracellular immunofluorescent staining. Cells were first surface stained, then fixed and incubated with anti-T-bet and anti-Eomes antibodies in permeabilization buffer (eBioscience, Cat# 00-5521) before analysis by flow cytometry.

### Statistical analysis

For the statistical analysis of the IVT and antigen presentation experiments, a two-tailed paired Student's t test was used. A P-value<0.05 was considered significant.

## Supporting Information

Figure S1 C57BL/6 mice were adoptively transferred with CFSE^+^CD8^+^ OT-1 cells, followed by intramuscular immunization two hours following transfer with recombinant EBNA1-SIIN-GFP adenoviral expression vector variants Ad-E1-GArN-SIIN-GFP, Ad-E1-GArM-SIIN-GFP, Ad-E1-SIIN-GFP, Ad-E1-ΔGA-SIIN-GFP or a control vector lacking SIINFEKL, Ad-E1-GFP. Mice were sacrificed and draining lymph node (DLN) cells were prepared on days 1, 2 or 3 post-infection. *Upper panels* demonstrate the overall proliferation of transferred CD8**^+^** OT-1 T cells on days 1, 2 and 3 from mice immunized with a high dose (1×10^8^ pfu/mouse) of each EBNA1-GFP expression vector. *Lower panels* demonstrate the expression of T cell activation markers CD44, CD62L and CD69 on days 1, 2 and 3 post-infection with each EBNA1-GFP expression vector at 1×10^8^ pfu/mouse.
(DOCX)

Table S1 Adenovirus transduction efficiencies for Ad-E1-GArN-GFP, Ad-E1-GArM-GFP, Ad-E1-GFP, Ad-E1-ΔGA-GFP in HEK293 cells at varying MOIs was estimated by measuring the percentage of GFP+ve cells and the GFP mean fluorescence intensity (MFI) by flow cytometry. Experiment 1 compares all four Ad-EBNA1-GFP variants at MOIs of 40 and 4. Experiment 2 compares Ad-E1-GArN-GFP and Ad-E1-GArM-GFP at MOIs of 50, 25 and 12.5.
(DOCX)
