# Supplementary material for: mRNA Structural Constraints on EBNA1 Synthesis Impact on In Vivo Antigen Presentation and Early Priming of CD8+ T Cells
Source: PLoS Pathog. 2014 Oct 9;10(10):e1004423. doi: 10.1371/journal.ppat.1004423 (PMC4192603; doi:10.1371/journal.ppat.1004423)
Supplement: Figure S1 — C57BL/6 mice were adoptively transferred with CFSE+CD8+ OT-1 cells, followed by intramuscular immunization two hours following transfer with recombinant EBNA1-SIIN-GFP adenoviral expression vector variants Ad-E1-GArN-SIIN-GFP, Ad-E1-GArM-SIIN-GFP, Ad-E1-SIIN-GFP, Ad-E1-ΔGA-SIIN-GFP or a control vector lacking SIINFEKL, Ad-E1-GFP. Mice were sacrificed and draining lymph node (DLN) cells were prepared on days 1, 2 or 3 post-infection. Upper panels demonstrate the overall proliferation of transferred CD8+ OT-1 T cells on days 1, 2 and 3 from mice immunized with a high dose (1×10^8^ pfu/mouse) of each EBNA1-GFP expression vector. Lower panels demonstrate the expression of T cell activation markers CD44, CD62L and CD69 on days 1, 2 and 3 post-infection with each EBNA1-GFP expression vector at 1×10^8^ pfu/mouse. (DOCX) [file ppat.1004423.s001.docx]

**SI Figure 1**
