# Supplementary material for: mRNA Structural Constraints on EBNA1 Synthesis Impact on In Vivo Antigen Presentation and Early Priming of CD8+ T Cells
Source: PLoS Pathog. 2014 Oct 9;10(10):e1004423. doi: 10.1371/journal.ppat.1004423 (PMC4192603; doi:10.1371/journal.ppat.1004423)
Supplement: Table S1 — Adenovirus transduction efficiencies for Ad-E1-GArN-GFP, Ad-E1-GArM-GFP, Ad-E1-GFP, Ad-E1-ΔGA-GFP in HEK293 cells at varying MOIs was estimated by measuring the percentage of GFP+ve cells and the GFP mean fluorescence intensity (MFI) by flow cytometry. Experiment 1 compares all four Ad-EBNA1-GFP variants at MOIs of 40 and 4. Experiment 2 compares Ad-E1-GArN-GFP and Ad-E1-GArM-GFP at MOIs of 50, 25 and 12.5. (DOCX) [file ppat.1004423.s002.docx]

| **Construct** | **MOI** | **GFP+ve cells** | **GFP MFI** |
| --- | --- | --- | --- |
| Ad-E1-GArN-GFP | 40 | 99.8 | 40,654 |
|  | 4 | 98.1 | 16,159 |
| Ad-E1-GArM-GFP | 40 | 99.9 | 65,488 |
|  | 4 | 99.7 | 32,977 |
| Ad-E1-GFP | 40 | 94 | 22,281 |
|  | 4 | 89.2 | 7,505 |
| Ad-E1-ΔGA-GFP | 40 | 95.3 | 34,590 |
|  | 4 | 88 | 8,292 |

**SI Table 1**

**Experiment 1**

**Experiment 2**

| **Constructs** | **MOI** | **GFP+ve cells** | **GFP MFI** |
| --- | --- | --- | --- |
| Ad-E1-GArN-GFP | 50 | 96.1 | 32,241 |
|  | 25 | 94 | 26,453 |
|  | 12.5 | 89.1 | 22,807 |
| Ad-E1-GArM-GFP | 50 | 97 | 54,021 |
|  | 25 | 95.5 | 44,547 |
|  | 12.5 | 92.1 | 34,896 |
